# The mediating role of executive function in the relationship between self-stigma and self-injury or suicidal ideation among men who have sex with men living with HIV

**DOI:** 10.3389/fpubh.2022.1066781

**Published:** 2023-01-09

**Authors:** Yixuan Li, Xueling Xiao, Yaqin Zhou, Xinyi Su, Honghong Wang

**Affiliations:** Xiangya School of Nursing of Central South University, Changsha, Hunan, China

**Keywords:** men who have sex with men, HIV, self-injury or suicidal ideation, self-stigma, executive function

## Abstract

**Background:**

Men who have sex with men (MSM) living with HIV suffer from psychosocial pressures and marginalization as a result of being HIV-positive and belonging to a sexual minority group, and self-injury or suicidal ideation are prevalent among this group. Studies have found that both perceived self-stigma and altered executive function is related to self-injury or suicidal ideation. However, the combined contribution of self-stigma and executive function to self-injury or suicidal ideation remains unclear, especially in MSM living with HIV. Therefore, this study is conducted to explore the mechanism of self-injury or suicidal ideation by hypothesizing that executive function plays a mediating role in the relationship between self-stigma and self-injury or suicidal ideation.

**Methods:**

We conducted a cross-sectional survey among 448 MSM living with HIV who were recruited in the HIV clinic of a tertiary general hospital in Changsha, China, from November 2021 to February 2022. A questionnaires survey was adopted to collect sociodemographic and disease-related information and data related to executive function (including working memory, inhibition, and task monitoring), self-stigma, and self-injury or suicidal ideation. Structural equation modeling and bootstrap testing were used to investigate the potential mediating role of executive function in the relationship between self-stigma and suicidal ideation.

**Results:**

The participants were aged 18–76 years. Those who had ever had self-injury or suicidal ideation accounted for 32.8% of the total. A higher level of self-stigma and poorer executive function were associated with more frequent self-injury or suicidal ideation (*p* < 0.01). The mediation model analysis showed a good fit (*x*^2^/*df* = 1.07, *p* = 0.381). The direct effect of self-stigma on self-injury or suicidal ideation (β = 0.346, *p* < 0.001) and the indirect effect of self-stigma *via* executive function (β = 0.132, *p* < 0.001) were significant, with the indirect effect accounting for 27.6% of the total effect.

**Conclusions:**

This study demonstrates that executive function mediates the relationship between self-stigma and self-injury or suicidal ideation among MSM living with HIV. It suggests that future studies targeting enhancing executive function and decreasing self-stigma may reduce self-injury or suicidal ideation among MSM living with HIV.

## Introduction

The number of men who have sex with men (MSM) living with HIV has steadily increased. Globally, the proportion of new HIV infections among MSM rose by 25% between 2010 and 2021 (1). Further, among the 1.5 million individuals newly infected with HIV in 2021, an estimated 21% were MSM (2). MSM living with HIV are a vulnerable and marginalized population. They suffer from psychosocial pressures as a result of being HIV-positive and belonging to a sexual minority group because of their homosexual identity (3). MSM living with HIV exhibit increased risks of self-injury or suicidal ideation owing to various issues including mental and physical health problems and risk-taking behaviors (4–6). Self-injury or suicidal ideation has a high possibility of leading to injury or death as ideation leads to actual behaviors (7), and the ideation of self-injury and suicide usually overlap and exist on a continuum which could be combined to predict actual suicide (8, 9). Concerningly, the incidence of self-injury or suicidal ideation among MSM living with HIV is 10.7–47.0% (10–12), which is 2.22 times higher than that among HIV-negative counterparts (13). Thus, we measured the self-injury or suicidal ideation as one combined outcome, and tried to understand its mechanism of self-injury or suicidal ideation among this specific population to enhance suicide prevention efforts.

Self-stigma, namely, the devaluation on oneself because of the perception of negative characteristics from the broader society (14), is considered an essential risk factor for self-injury or suicidal ideation in MSM living with HIV. In both developed and developing settings, MSM, as a sexual minority group, suffer unfairness and rejection from their peers and face a high risk of losing their jobs or experiencing barriers to accessing health care, all of which can increase their self-stigma (15, 16). For MSM living with HIV report even higher levels of self-stigma, reflecting their worse situation (17, 18). MSM living with HIV suffer from higher self-stigma because of the prejudice of the public toward them, such as through beliefs that such individuals engaged in immoral sexual behaviors (e.g., having condom-less anal sex after substance use or having multiple sexual partners) (16). High levels of self-stigma can have various negative consequences, including experiences of prejudice and social isolation in daily life and a fear of seeking healthcare (19). These findings are particularly troublesome given that being isolated or lacking healthcare prevent these individuals from getting professional HIV treatment and necessary mental health support they need (20). Ultimately, this combination of factors marginalizes such individuals and drives them into a state of hopelessness (21), which can lead to self-injury, suicidal ideation, or even suicide (22). A previous study showed that MSM living with HIV and perceiving self-stigma were 2.4 times more likely to have suicidal ideation than those without self-stigma (23).

Executive function also has a potential impact on self-injury or suicidal ideation among MSM living with HIV. Executive function is a high-level cognitive process that facilitates individuals to behave reasonably, purposefully, and thoughtfully (24). Theoretically, the core aspects of executive function, including working memory, inhibition, and task monitor, are associated with individuals' behaviors and intentions (25). Deficits in executive function can result in misunderstandings and inaccurate responses to internal and external environmental challenges; consequently, individuals may be easily overwhelmed and may engage in self-injury or suicidal ideation (26). Previous studies found executive function impairments among the HIV-negative population with suicidal ideation or suicidal behavior (27–29). Although limited evidence is available for HIV-positive individuals, one previous study suggested that suicidal ideation in this population might be a reflection of executive function deficits (30). Moreover, executive function impairment has been widely reported among HIV-positive individuals, as the virus can invade the frontal lobe of the brain, which is the area that controls executive function (31–33). Therefore, the connection between executive function and self-injury or suicidal ideation needs to be examined among MSM living with HIV who are vulnerable to having impaired executive function.

In addition, self-stigma is associated with executive function. It has been reported that self-stigma can have a direct effect on cognitive performance as it can reduce reasoning ability (34), thus influencing the executive function. Sexual minorities often report both impaired executive function and a high level of self-stigma (35–38). This association was also demonstrated among people living with HIV. Two studies revealed that HIV-related stigma is a significant predictor of poor performance on cognitive tests and may have long-lasting effects on brain function, especially executive function (39, 40). Moreover, a previous study investigating 70 MSM living with HIV reported that individuals with lower HIV-related self-stigma performed better in working memory, a core aspect of executive function (41). In this regard, self-stigma, especially that related to HIV, may be associated with deficits in executive function.

However, how executive function affects the relationship between self-stigma and self-injury or suicidal ideation is unclear as executive function impairment is associated with both. Furthermore, this correlation needs to be explored in MSM living with HIV as this vulnerable population faces all three difficulties: impaired executive function, severe self-stigma, and a high level of self-injury or suicidal ideation. Thus, this study is conducted to investigate the mechanism of self-injury or suicidal ideation among MSM living with HIV by hypothesizing that executive function is negatively associated with suicidal ideation and mediates the relationship between self-stigma and self-injury or suicidal ideation. The study could provide evidence for the further understanding and development of interventions for the prevention of self-injury and suicidal ideation among MSM living with HIV.

## Methods

### Setting and participants

The present cross-sectional study was conducted from November 2021 to February 2022 in Changsha, China. We recruited participants in an HIV clinic of a tertiary general hospital in Changsha, where the annual outpatient volume is approximately 35,000 visits. The eligibility criteria were individuals (1) aged 18 years or older; (2) born as males; (3) self-reporting as being homosexual, bisexual, or having sex with men; and (4) diagnosed with HIV (as confirmed by diagnosis report). Individuals who self-reported having a history of brain injury or brain surgery, or who were unable to finish the survey for any intellectual or physical reason, were excluded.

### Recruitment and data collection

Participants were recruited when they came to the HIV clinic to access health services. Four trained research assistants approached and screened 724 males for eligibility to participate. Of these, 58 were excluded because they did not have a confirmed HIV diagnosis (*n* = 55) or were unable to complete the questionnaire (*n* = 3). Around 30% (218/724) of eligible MSM living with HIV rejected to participate in this study because they were not interested or did not have time. Finally, 449 participants completed the survey.

Participation was on a voluntary basis. The HIV-infected status was confirmed through each participants' diagnosis report. After obtaining informed consent from the participants, the research assistants provided a brief explanation of how to complete the survey. Participants were then invited to answer the questionnaire in a quiet office with good privacy. Every completed questionnaire was deposited in a locked storage box by the research assistants. One incomplete questionnaire was excluded, resulting in a total of 448 valid questionnaires being included in this study.

### Measures

The questionnaire consisted of four parts to collect sociodemographic and disease-related information, and data related to self-injury or suicidal ideation, executive function, and self-stigma.

#### Sociodemographic and disease-related information

Sociodemographic characteristics included age, education level, occupational status, marital status, and monthly income. Disease-related information included HIV duration (time elapsed since the HIV diagnosis), latest CD4 count (cells/μl), and HIV viral load, all of which were obtained from medical records. An HIV viral load lower than 20 copies per ml was categorized as target not detected (TND). Those without CD4 count and HIV viral load data were defined as unknown.

#### Self-injury or suicidal ideation

Self-injury or suicidal ideation was assessed using a single item: “Over the last 2 weeks, how often have you been bothered by thoughts that you would be better off dead or hurting yourself in some way?” This method has been used in several studies (42–44) and has proven validity in assessing individuals' self-injury or suicidal ideation. The response options ranged from 0 to 3 (0 = not at all, 1= several days, 2 = more than half the days, 3 = nearly every day). In this study, self-injury or suicidal ideation was categorized as a dichotomous variable by combining those who chose 1, 2, or 3 as “yes” and those who chose 0 as “no.”

#### Executive function

Executive function was assessed using the Chinese version of the Behavior Rating Inventory of Executive Function—Adult Version, a widely used 3-point Likert scale with perfect psychometric characteristics (45). This scale includes eight dimensions, each of which measures one aspect of executive function (46). Specifically, we measured three core dimensions of executive function—inhibition (e.g. “I have difficulty in waiting in line”), working memory (e.g. “I am prone to forgetting instructions”), and task monitoring (e.g. “I do not check for mistakes in my work”)—which contained 8, 8, and 6 items respectively. Every item was scored from 1 to 3 (1= never, 2 = sometimes, 3 = always). The possible range were 8–24, 8–24, and 6–18 for the domain of inhibition, working memory, and task monitoring, respectively, and a higher score in a particular dimension reflected poorer executive function in this domain.

#### Self-stigma

Self-stigma among MSM living with HIV was measured by a brief stigma scale containing 10 items for HIV-positive individuals. This scale is widely used to assess the HIV-related stigma of people living with HIV and is known to show good reliability and validity (47). This scale includes four domains of self-stigma: personalized stigma, disclosure concerns, negative self-image, and public attitudes. For example, one of the items had asked “I feel that I am not as good a person as others”, and the answer rated on a 4-point Likert scale ranged from “strongly disagree” to “strongly agree.” The total score ranged from 10 to 40, and a higher score reflected a higher level of self-stigma.

### Data analysis

All data were double input in EpiData Version 3.1 to ensure accuracy and integrity. The descriptive and correlation analyses were conducted in SPSS Version 22.0. Percentages were used to summarize categorical variables. For continuous variables, a normality test was performed first, and the means (standard deviations, SDs) and medians (interquartile, IQR) were adopted accordingly. Pearson's correlation test was used to identify the association between continuous variables, and a point-biserial correlation test was used to verify the association between continuous variables and the binary variable.

The mediation model was explored using Mplus Version 7.0. The Mplus code was based on the original PROCESS diagrams by Andrew Hayes (48). We modified model number 4d, which showed an example of how to handle a dichotomous outcome. We used bootstrap testing with 1,000 resamples to calculate the 95% confidence intervals (CIs). It indicated a significant direct and indirect effect if the 95% CI excluded zero. We tested the unadjusted mediation model and then the adjusted model with the confounding variables being controlled. Variables that showed a significant effect on the mediating or outcome variable after monofactor analysis were defined as confounding variables (49). In this mediation model, we found no confounders of self-injury; however, age, education level, and occupational status were identified to be confounding variables of executive function. Thus, these three variables were controlled in the adjusted model. Statistical test volumes including *x*^2^/*df*, root mean square error of approximation (RMSEA), comparative fit index (CFI), and Tucker–Lewis index (TLI) were used to evaluate the goodness-of-fit of the model. As suggested by Dexin Shi (50), RMSEA should be lower than 0.08, CFI and TLI should be no < 0.90, and *x*^2^/*df* should not be higher than 2.00.

### Ethics consideration

This study was approved by the Institutional Review Board of the Xiangya School of Nursing, Central South University. Participants who reported having self-injury or suicidal ideation were directed to a psychological clinic in the city for further treatment and guidance. We provided a compensation of Renminbi 50 Yuan (equaling to USD $6.95) to each participant. No identifying information was collected in this study, and only research group members had access to the data.

## Results

### Characteristics of the study sample

A total of 448 valid questionnaires were included in this study, and the sample characteristics are shown in Table 1. The participants were aged 18–76 years, with a median age of 30 years. Only around a quarter (26.1%) were married or partnered. The HIV durations were 1–210 months, with a median duration of 47.5 months. A majority of the participants (*n* = 401, 89.5%) had a CD4 count above 200 cells/μl, and around three-quarters (*n* = 332, 74.1%) were in TND status.

**Table 1:** Differences in self-injury or suicidal ideation according to general characteristics (*n* = 448).

**Variables**	***n* (%)/Median/IQR (25%, 75%)**	**Self-injury or suicidal ideation**		**OR (95% CI)**	***p***
		**Yes (*****n** =* **147)**	**No (*****n** =* **301)**		
		***n*** **(%)/median/IQR (25%, 75%)**	***n*** **(%)/median/IQR (25%, 75%)**		
Age (years)	30 (25, 34)	29 (24, 34)	30 (25, 34)	0.98 (0.96–1.01)	0.12
**Education level**					
High school or lower	73 (16.3)	24 (32.9)	49 (67.1)	Ref	
Vocational education	144 (32.1)	43 (29.9)	101 (70.1)	0.87 (0.47–1.59)	0.65
University or higher	231 (51.6)	80 (34.6)	151 (65.4)	1.08 (0.62–1.89)	0.78
**Occupational status**					
Unemployed	89 (19.9)	34 (38.2)	55 (61.8)	Ref	
Employed	359 (80.1)	113 (31.5)	246 (68.5)	0.74 (0.46–1.20)	0.23
**Marital status**					
Married/partnered	117 (26.1)	32 (27.4)	85 (72.6)	Ref	
Separated/single/widowed	331 (73.9)	115 (34.7)	216 (65.3)	1.41 (0.88–2.25)	0.14
**Monthly income (RMB)**					
≤ 2,000	123 (27.5)	49 (39.8)	74 (60.2)	Ref	
2,000–4,000	114 (25.4)	35 (30.7)	79 (69.3)	0.89 (0.52–1.54)	0.70
≥4,000	211 (47.1)	63 (29.9)	148 (70.1)	1.18 (0.59–2.35)	0.63
HIV duration (months)	47.5 (20.0)	43.0 (20.0)	51.0 (21.0)	1.00 (0.99–1.01)	0.17
**Latest CD4 count**					
≤ 200	25 (5.6)	10 (40.0)	15 (60.0)	Ref	
200–500	190 (42.4)	58 (30.5)	132 (69.5)	0.66 (0.28–1.55)	0.34
≥500	211 (47.1)	70 (33.2)	141 (66.8)	0.74 (0.32–1.74)	0.49
Unknown	22 (4.9)	9 (40.9)	13 (59.1)	1.04 (0.32–3.34)	0.95
**Viral load**					
TND status	332 (74.1)	106 (31.9)	226 (68.1)	Ref	
Not TND status	45 (10.0)	14 (31.1)	31 (68.9)	0.96 (0.49–1.88)	0.91
Unknown	71 (15.9)	27 (38.0)	44 (62.0)	1.30 (0.77–2.23)	0.32

RMB, Renminbi (Chinese Yuan); TND status, target not detected (HIV viral load < 20 copies per ml); IQR, interquartile; OR, odds ratio; 95%CI, 95% confidence interval.

### Prevalence of self-injury or suicidal ideation

Overall, 32.8% (147/448) of the participants reported self-injury or suicidal ideation. Specifically, around one-fifth (21.7%, 97/448) of the participants reported that they had thought of suicide or hurting themselves in some way for several days over the past 2 weeks; 8.3% (*n* = 37) had this ideation more than half of the days; and 13 participants reported that they had thought of self-injury or suicide nearly every day. However, there was no statistically significant difference in self-injury or suicidal ideation based on general characteristics (see Table 1).

### Self-stigma and executive function

As shown in Table 2, the mean score for self-stigma was 28.46 (SD 5.97), exceeding 70% of the possible range. The scores for the three core executive functions, namely, inhibition, working memory, and task monitoring, were all at an intermediate level, with medians of 12, 11, and 9, respectively.

**Table 2:** Correlations for the relationships between executive function (inhibition, working memory, task monitor), self-stigma, and self-injury or suicidal ideation.

	**Mean/median**	**SD/IQR**	**Possible range**	**Actual range**	**1**	**2**	**3**	**4**	**5**
1. Inhibition^a^	12	10	8–24	8–24	1				
2. Working memory^a^	11	9	8–24	8–24	0.624^**^	1			
3. Task monitor^a^	9	7	6–18	6–18	0.569^**^	0.699^**^	1		
4. Self-stigma^a^	28.46	5.97	10–40	12–40	0.322^**^	0.357^**^	0.343^**^	1	
5. Self-injury or suicidal ideation^b^	NA	NA	NA	NA	0.261^**^	0.317^**^	0.225^**^	0.396^**^	1

^a^Pearson's correlation test.

^b^Point-biserial correlation test.

^**^P < 0.01.

IQ, interquartile; SD, standard deviation; NA, not applicable.

### Relationships between executive function, self-stigma and self-injury or suicidal ideation

Self-injury or suicidal ideation was significantly associated with inhibition (*r* = 0.261, *p* < 0.01), working memory (*r* = 0.317, *p* < 0.01), and task monitoring (*r* = 0.225, *p* < 0.01; Table 2). Furthermore, suicidal ideation had a positive correlation with self-stigma (*r* = 0.396, *p* < 0.01), indicating that higher self-stigma was associated with more frequent self-injury or suicidal ideation. Self-stigma was also related to three core executive functions, with the correlation coefficient ranging from 0.322 to 0.357 (*p* < 0.01).

### Mediation analysis

**Figure 1:**
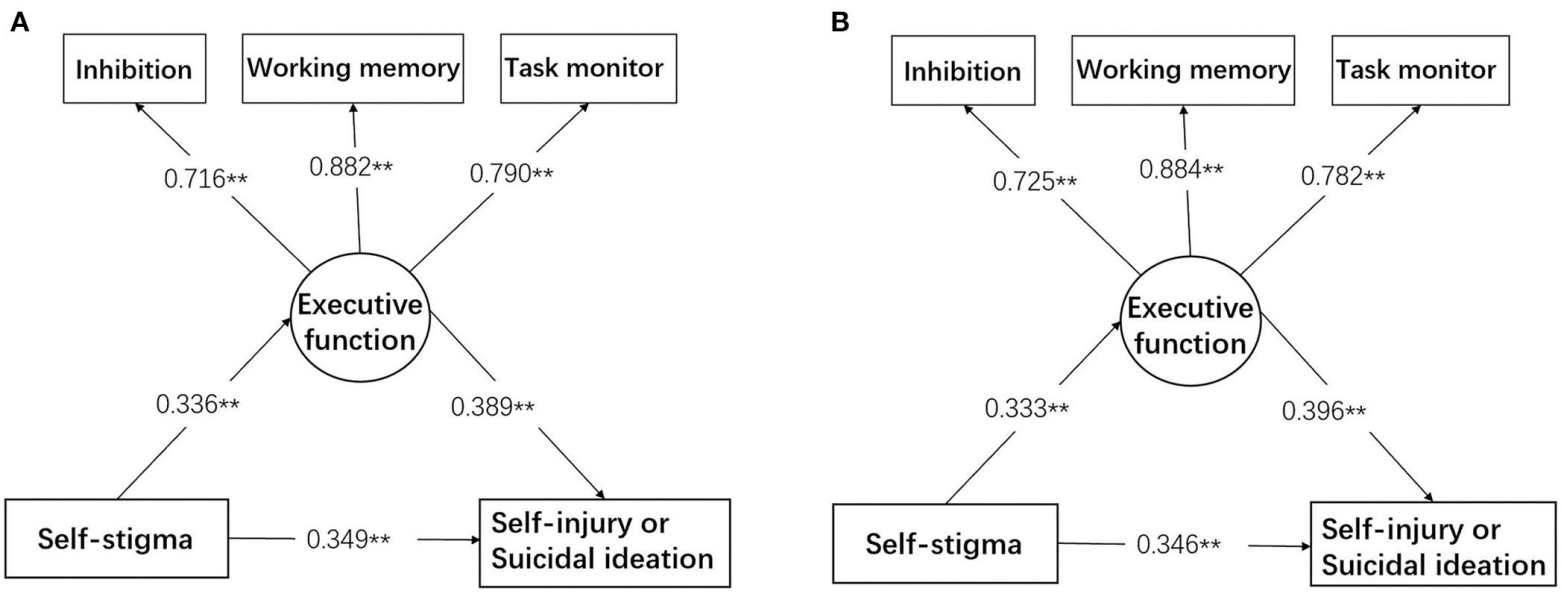
Mediation model in which the relationship between self-stigma and self-injury or suicidal ideation was mediated by the three core executive functions, namely, inhibition, working memory, and task monitor. **(A)** Shows the unadjusted model. **(B)** Shows the model adjusted by age, education level, and occupational status. **p < 0.001.

The results of the mediation analysis are shown in Table 3 and Figure 1. In the unadjusted model, the direct effect of self-stigma on self-injury or suicidal ideation was statistically significant (β = 0.349, *p* < 0.001), as was the indirect effect (β = 0.131, *p* < 0.001). The indirect effect accounted for 27.3% of the total effect.

**Table 3:** Parameters of the mediated model of self-stigma and self-injury or suicidal ideation mediated by executive function.

	**Path**	**Std.β**	**95% CI (bootstrap test)**	**Model fit index**			
				*x*^2^/*df* **(P)**	**RMSEA**	**CFI**	**TLI**
Model 1	Self-stigma—self-injury or suicidal ideation	0.349^**^	0.224–0.451	1.42 (*p* = 0.223)	0.031	0.996	0.990
	Self-stigma—executive function—self-injury or suicidal ideation	0.131^**^	0.088–0.191				
Model 2	Self-stigma—self-injury or suicidal ideation	0.346^**^	0.219–0.450	1.07 (*p* = 0.381)	0.012	0.998	0.997
	Self-stigma—executive function—self-injury or suicidal ideation	0.132^**^	0.088–0.192				

Model 1 is unadjusted. Model 2 is adjusted by age, education level, and occupational status.

^**^P < 0.01.

Std. β, standardized estimate; CI, confidence interval; *df*, degree of freedom; RMSEA, root mean square error of approximation; CFI, comparative fit index; TLI, Tucker–Lewis index.

The adjusted model with age, education level, and occupational status being controlled (Figure 1B) showed a higher proportion of indirect effect (27.6%) compared to the case of the unadjusted model. The direct effect of self-stigma on self-injury or suicidal ideation decreased to 0.346 (*p* < 0.001), and the indirect effect increased to 0.132 (*p* < 0.001).

The three significant variables of inhibition, working memory, and task monitor well represented the executive function, with factor loadings of 0.716–0.882 (*p* < 0.001). The mediation model also demonstrated good fit information, and the fit index was better when adjusted for confounding variables (*x*^2^/*df* = 1.07, *p* = 0.381, RMSEA = 0.012, CFI = 0.998, TLI = 0.997).

## Discussion

This study explored the relationships between self-injury or suicidal ideation, self-stigma, and executive function among MSM living with HIV. Our sample showed high levels of both self-injury or suicidal ideation and self-stigma, indicating that the participants were suffering from a heavy psychosocial burden. Overall, in line with our hypothesis, poorer executive function was associated with a higher level of self-injury or suicidal ideation, and the relationship between self-injury or suicidal ideation and self-stigma among MSM living with HIV was partly mediated by their executive function.

Around 30% of MSM living with HIV reported self-injury or suicidal ideation in this sample, which was consistent with the results of previous studies in China (23, 51). The proportion was much higher than that in countries with more inclusive sexual attitudes, such as the US (3.2%) and Spain (15.3%) (52, 53). The high prevalence of self-injury or suicidal ideation may be attributed to the traditional sexual values that are deeply rooted in Chinese society, namely, that homosexuality is unacceptable to the majority of Chinese people (54). In China, individuals living with HIV are considered to be reckless individuals, and they suffer from stigma and discrimination from the public and even their family members or friends, which can increase the likelihood of suicide (55). Importantly, the incidence of self-injury or suicidal ideation among MSM living with HIV is higher than that among general MSM (18.3%) or heterosexual HIV-positive individuals (22.5%) (56, 57). This may be explained by the fact that MSM living with HIV are under the dual pressure of being HIV-positive and belonging to a sexual minority group, which could drive them to hurt themselves. As stated in the Minority Stress Theory (58), MSM living with HIV perceived a culturally sanctioned and categorically ascribed inferior status, resultant prejudice, and discrimination. The cumulative experiences were gradually internalized to the internal stress which worsened their survival situation and may lead to the appearance of self-injury or suicidal ideation. Thus, there is a more urgent need to increase support for MSM living with HIV compared to that for general HIV-positive individuals or other sexual minorities.

As we hypothesized, executive function was negatively associated with self-injury or suicidal ideation among MSM living with HIV. This finding was aligned with previous studies that focused on mood disorder samples, which showed that impaired executive function could be a risk factor of suicidality, because those with suicidal ideation showed reduced structural connectivity among the frontal-subcortical circuits, including in the regions associated with executive function (59, 60). In addition, executive function deficit was considered a candidate endophenotype for genetics research on suicidal behavior (61). Individuals with executive function deficits find it difficult to be reflective, are likely to act impulsively (62), are unable to reason based on factual information and evaluation (63), and lack rational thinking (64–66). It is therefore understandable that for some individuals with impaired executive function, committing suicide seems to be the only option when they face stressful life events, because they struggle to reflect, reason, and think rationally about their situation and are likely to make an impulsive decision.

Our results show that executive function partially mediated the relationship between self-stigma and self-injury or suicidal ideation among MSM living with HIV. Moreover, the fact that executive function accounted for 27.6% of this association indicates that executive function plays an important role in it. Perceptions of stigma, especially HIV-related self-stigma, drive individuals to avoid social activities and isolate themselves (67). Social isolation may have a negative effect on brain structures, which is reflected in low executive function performance (68). A possible explanation for the negative effect is that those with a high level of self-stigma suffer from chronic stress caused by people around them or even themselves (17). When they are stressed, they are more concerned about being judged negatively than about thinking rationally to reach their goals (69). Gradually, they lose their goal-oriented behavioral ability, eventually leading to retrogressive executive function. In turn, MSM living with HIV who have executive function deficits are thought to have difficulty in thinking about long-term outcomes and usually focus on the current dilemma (70, 71), which might culminate in despair and lead to self-injury or suicidal ideation as the only solution they recognize. In light of this, it is necessary to think about the impact of executive function when analyzing self-injury or suicidal ideation and self-stigma among MSM living with HIV.

To the best of our knowledge, this was the first study conducted among MSM living with HIV to explore the mediating role of executive function in the relationship between self-injury or suicidal ideation and self-stigma. This research provides a theoretical basis for and empirical evidence of the mechanism of self-injury or suicidal ideation among this population, thus enabling a better understanding. Moreover, it provides a possible route for future intervention development to decrease suicidal behavior among MSM living with HIV. This study implies that strategies for improving executive function and simultaneously eliminating self-stigma may achieve an optimal outcome in reducing self-injury or suicidal ideation or behavior among MSM living with HIV. Existing studies have demonstrated the effectiveness of self-stigma avoidance methods such as increasing the awareness of mental health condition and strengthening self-regulation skills (72, 73). Further, executive function training, such as helping them identify their own motivating goals or activate intentional self-regulation in stressful situations, showed effectiveness in changing behaviors such as sedentary activity, unhealthy eating and inability to sit still (74, 75). These abovementioned strategies could be adopted among MSM living with HIV to reduce self-injury or suicidal ideation.

Inevitably, there were some limitations in this study. First, the cross-sectional design limited the study's ability to confirm the causal associations between variables. A longitudinal study should be conducted to obtain a deeper understanding of these relationships. Second, this study only explored three core executive functions instead of all the domains. Therefore, the generalization of the results related to executive function and self-injury or suicidal ideation should be treated with caution. Third, our sample only included MSM living with HIV in Hunan. A sample from other regions with various economic and cultural backgrounds may have generated different results, because the public attitudes toward MSM and HIV are deeply associated with the local culture and social norms.

## Conclusion

The MSM living with HIV suffer from a high level of self-injury or suicidal ideation, and executive function is negatively associated with this. Importantly, the correlation between self-stigma and self-injury or suicidal ideation is partially mediated by executive function among this specific population. Therefore, interventions that target promoting executive function and reducing self-stigma may work optimally to reduce self-injury and suicidal ideation in this population.

## Data availability statement

The original contributions presented in the study are included in the article/Supplementary material, further inquiries can be directed to the corresponding author.

## Ethics statement

The studies involving human participants were reviewed and approved by the Institutional Review Board of the Nursing School of Xiangya, Central South University. The patients/participants provided their written informed consent to participate in this study.

## Author contributions

XX designed this study. YL wrote the manuscript and performed the statistical analyses. YZ and XS contributed to data interpretation. XX and HW edited and revised the manuscript for important intellectual content and gave scientific advice. All authors contributed to this article and approve the final submission.
