# Supplementary material for: The mediating role of executive function in the relationship between self-stigma and self-injury or suicidal ideation among men who have sex with men living with HIV
Source: Front Public Health. 2023 Jan 9;10:1066781. doi: 10.3389/fpubh.2022.1066781 (PMC9869120; doi:10.3389/fpubh.2022.1066781)
Supplement: Supplementary file 1 [file Data_Sheet_1.pdf]

Bootstrap Test results in Model 1

MSM-EF-Model1 - 记事本
文件(F) 编辑(E) 格式(O) 查看(V) 帮助(H)

Total	0.062	0.067	0.072	0.092	0.111	0.116	0.124
Total indirect	0.014	0.017	0.018	0.025	0.035	0.036	0.041

Specific indirect

Y							
M							
X	0.014	0.017	0.018	0.025	0.035	0.036	0.041

Direct

Y							
X	0.033	0.041	0.044	0.067	0.086	0.091	0.098

CONFIDENCE INTERVALS OF STANDARDIZED TOTAL, TOTAL INDIRECT, SPECIFIC INDIRECT, AND DIRECT EFFECTS

STDYX Standardization

	Lower .5%	Lower 2.5%	Lower 5%	Estimate	Upper 5%	Upper 2.5%	Upper .5%
Effects from X to Y							
Total	0.337	0.364	0.384	0.480	0.557	0.571	0.599
Total indirect	0.075	0.088	0.092	0.131	0.183	0.191	0.212
Specific indirect							
Y							
M							
X	0.075	0.088	0.092	0.131	0.183	0.191	0.212
Direct							
Y							
X	0.186	0.224	0.239	0.349	0.436	0.451	0.477

Bootstrap Test results in Model 2

MSM-EF-Model2 - 记事本
文件(F) 编辑(E) 格式(O) 查看(V) 帮助(H)

Effects from X to Y

Total	0.061	0.067	0.071	0.092	0.112	0.117	0.124
Total indirect	0.014	0.016	0.018	0.025	0.035	0.037	0.041

Specific indirect

Y							
M							
X	0.014	0.016	0.018	0.025	0.035	0.037	0.041

Direct

Y							
X	0.032	0.040	0.043	0.067	0.086	0.090	0.097

CONFIDENCE INTERVALS OF STANDARDIZED TOTAL, TOTAL INDIRECT, SPECIFIC INDIRECT, AND DIRECT EFFECTS

STDYX Standardization

	Lower .5%	Lower 2.5%	Lower 5%	Estimate	Upper 5%	Upper 2.5%	Upper .5%
Effects from X to Y							
Total	0.338	0.363	0.382	0.478	0.555	0.569	0.596
Total indirect	0.075	0.088	0.094	0.132	0.181	0.192	0.209
Specific indirect							
Y							
M							
X	0.075	0.088	0.094	0.132	0.181	0.192	0.209
Direct							
Y							
X	0.175	0.219	0.236	0.346	0.434	0.450	0.474
